# Prevention is political: political party affiliation predicts perceived risk and prevention behaviors for COVID-19

**DOI:** 10.1186/s12889-022-12649-4

**Published:** 2022-02-14

**Authors:** Marc T. Kiviniemi, Heather Orom, Jennifer L. Hay, Erika A. Waters

**Affiliations:** 1 University of Kentucky, 151 Washington Avenue, Lexington, KY 40536 USA; 2 University at Buffalo, SUNY, Buffalo, USA; 3 Memorial Sloan Kettering Cancer Center, New York City, USA; 4 Washington University in Saint Louis School of Medicine, St. Louis, USA

**Keywords:** Risk perception, Political affiliation, Preventive behaviors, COVID-19, SARS-CoV-2

## Abstract

**Background:**

Many US politicians have provided mixed messages about the risks posed by SARS-CoV-2/COVID-19 and whether and to what extent prevention practices should be put in place to prevent transmission. This politicization of the virus and pandemic may affect individuals’ risk perceptions and willingness to take precautions. We examined how political party affiliation relates to risk perception for one’s own and other people’s likelihood of SARS-CoV-2 infection/COVID-19 illness.

**Methods:**

We surveyed members of a nationally-representative, probability-sampling based survey panel (*N* = 410) to examine their risk perceptions, precautionary behaviors, and political party affiliation.

**Results:**

The more strongly one identified as a Republican, the less risk one perceived to oneself from SARS-CoV-2/COVID-19 and the less risk one perceived other people faced. Moreover, those identifying as more strongly Republican engaged in fewer preventive behaviors.

**Conclusions:**

This differential response may affect virus transmission patterns and poses a considerable challenge for health communications efforts.

## Background

As of November 23, 2021, there were over 43.6 million SARS-CoV-2 infections in the US [1, 2] and more than 700,000 COVID-19 deaths [1, 3]. Both the infection and the death rates likely substantially underestimate the true population impact given that there are nearly 300,000 excess deaths in the US since the start of the pandemic [4, 5]. In addition to the mortality burden, there are long-term cardiac, respiratory, and other health consequences for COVID-19 patients [6, 7]. In many areas of the US, infection rates have shifted over time with changes in policy-mandated preventive strategies, decreasing when prevention-oriented policies were in place and then increasing again as prevention strategies were eased [8].

A centerpiece of public health strategies to control infectious disease spread is either recommending or mandating that members of the public engage in protective behavior to prevent disease transmission. In the case of COVID-19, staying 6 ft apart, wearing masks, working remotely when possible, avoiding public gatherings, and other strategies have been endorsed and communicated to the public by CDC, FEMA, WHO, and other national and international public health agencies [9–11]. These preventive strategies are effective at slowing the rate of COVID-19 infection [12–14]. However, for infectious disease prevention behaviors to be effective, they must be undertaken consistently by a sufficient proportion of the population to slow transmission [15–17].

*Social and Cultural Influences on Construction of Risk Perceptions and Preventive Behaviors.*

Most theoretical and empirical treatments of risk perception focus on individual cognitive and reasoning processes as the key determinant of a person’s risk perceptions [18, 19]. In contrast, the social amplification of risk framework [20, 21] describes the process by which scientific evidence, the ways in which people obtain information (e.g., news media), and political and cultural forces shape how individuals interpret and prioritize health risk information. According to this framework, perceptions of risk can be amplified or attenuated through social processes that influence: 1) the availability of risk information (e.g., via media and political sources) and 2) society’s response to the information (e.g., discourse about the veracity of the risk information in the media and interactions with cultural and peer groups [20]).

Influential communicators such as social/activist organizations and opinion leaders among social groups or organizations are key sources of information and can influence discourse surrounding risk information [21]. For example, political groups, parties, and leaders are prominent examples of influential communicators who may shape how their members and affiliates interpret risk information. These amplification and attenuation processes through influential communicators likely contribute to an alignment between, on one hand, people’s political affiliation and their underlying values and, on the other hand, their perceived risk.

During the pandemic, politicians in multiple countries have sought to control the amount and kind of information the public received about COVID-19 risk as well as actively disputing scientific discourse about the risk. Residents of several countries received messages from political leaders that minimized risk and raised doubt about preventive strategies. For example, the Prime Minister of Great Britain, Boris Johnson, publicly announced that he would not engage in social distancing and, specifically, would continue to shake hands [22] less than a week before the British government began to plan policy strategies to prevent transmission [23]. In Brazil, President Jair Bolsonaro made public statements that minimized the perception that the virus posed a risk, comparing it to the flu [24] and actively arguing against preventive policy strategies that were being put in place in Brazilian cities [25, 26]. Similar minimizing and contradictory statements can be found from the leaders of other countries [27].

In the United States, the country of focus in the current study, then President Donald Trump regularly made public statements that downplayed the threat posed by the virus [28, 29] in terms of both severity, comparing it to the flu [30], and the number of cases and deaths [31]. President Trump also downplayed the importance of preventive actions to protect against transmission, including arguing against mask mandates [32], pushing for early easing of stay at home and business closure orders [33], and holding public gatherings in spite of social distancing policies in place [34]. The President admitted to being motivated to downplay the risk despite receiving clear warnings about the severity and seriousness of the spread of COVID-19 ([35] p. xviii).

In addition to risk perception, individuals’ decision making about COVID-19 prevention behaviors has taken place in the context of a complex, saturated, fast-moving information environment, with multiple and sometimes contradictory messages from traditional media, social media, and government messaging [36]. There is evidence that political messaging has an influence on behavior for individuals who support the politician conveying the messaging. Specifically, a study of the impact of President Trump’s anti-vaccination messaging found that the exposure to the messages negatively impact vaccination engagement intentions, but only on the part of voters who voted for him [37].

Based on the social amplification of risk framework, one would expect that the selective communication of risk information by politicians and the conflating of politics and public health in media messaging would influence the public’s perceptions of risk and their decision making about behavioral strategies to mitigate risk. Moreover, given the current US phenomenon where some news media outlets provide partisan lenses on issues, one would expect this effect to be exacerbated as news media “amplify” the messaging about risk perception [38]. Thus, one would predict that political affiliation would, by affecting exposure, attention, processing, and response to SARS-CoV-2/COVID-19 messages, influence risk perception and behavior.

Given the empirical evidence and plausible, theory-derived mechanisms for the role of social amplification in risk perception and decision making about risk reduction messages, we hypothesize that political party affiliation will relate to engagement in preventive measures for SARS-CoV-2/COVID-19, such that – given the predominant minimization themes in President Trump’s statements, those who identify more strongly as Republicans will be less likely to engage in preventive measures and will perceive less risk relative to those who identify more strongly as Democrats.

We examined whether Americans’ political party identification is related to perceived risk of SARS-CoV-2 infection, the severity of COVID-19 illness, and engagement in a range of preventive behaviors. Although there have been examinations of how political affiliation relates to support for preventive measures against COVID-19 (e.g., policy measures to restrict gatherings, mask wearing), our approach adds to this literature in two ways. First, we utilize a nuanced, quasi-continuous assessment of strength of party affiliation, allowing for an understanding of how strength of political beliefs affect responses to COVID-19. Second, we examine the effects of political partisanship on risk perceptions for COVID-19 infection in addition to support for preventive measures, adding to the understanding of how politicians’ risk minimizing messages might affect individuals’ perceptions of the risks posed by COVID-19.

## Methods

We conducted a cross-sectional, population-representative national survey of US adults. The study was reviewed and determined exempt by the University of Kentucky Institutional Review Board.

### Participants

A random sample of 410 members of the Ipsos KnowledgePanel [39] were surveyed by Ipsos. KnowledgePanel is a survey panel recruited using probability-based sampling techniques. The sampling frame for the panel is therefore population representative of US adults aged 18 and older and non-institutionalized. The survey was conducted over 6 days in mid-June 2020. Given the novelty of COVID-19 risk perceptions and behaviors at the time, there were not existing data on which to base an effect size estimate. Therefore, the necessary sample size was calculated to allow for an 80% power to detect a “small effect” (Cohen’s f^2^ > = 0.02) for the relation between party affiliation an outcomes.

### Measures

#### Political affiliation

Political affiliation was assessed using the methods of the American National Election Studies [40]. Participants were first asked to indicate whether they identified as a Democrat, Republican, Independent, another party, or no preference. Those indicating Democrat or Republican were then asked a follow-up question in which they were asked whether their party affiliation was “strong” or “not very strong”. Those indicating independent were then asked whether they more strongly identified with the Republican or the Democratic party. These two questions were used to create a 6-point party affiliation measure ranging with levels of Strong Republican, Weak Republican, Independent-Lean Republican, Independent Lean Democrat, Weak Democrat, and Strong Democrat. Only 11 of the 410 respondents reported no preference or another party affiliation (2.6% of the sample, a percentage consistent with other nationally-representative assessments of party affiliation [41]). Given their very small number, these respondents were not included in the political affiliation analyses.

#### Risk perception

We assessed risk perception for two different referents – participants’ perception of their *own* risk for SARS-CoV-2 infection and their perception of the risk for an average person in the US (*other* risk). We asked about four different components of risk perception that have all been shown to be important in behavioral adoption. First, we asked participants to report perceptions of the *absolute risk* for infection using a measure modified from the Health Information National Trends Survey [42, 43] (“How likely are you to get COVID-19 in the next 6 months?”). Second, we asked participants to report the how much *fear* they experienced concerning the virus [44] (“How afraid are you of getting COVID-19 in the next 6 months?”). Third, we asked participants to provide a gist-based assessment of how likely they *felt* infection was to happen to them [45, 46] (“How easily do you feel you could get COVID-19 in the next 6 months?”). Fourth and finally, we asked participants to report how severe infection would be were it to occur to them (Modified from [47]; “If you were to get COVID-19 in the next 6 months, how serious would it be?”). Each risk perception question was assessed with a four-point scale, with a score of 1 indicating the lowest level of perceived risk and a score of 4 indicating the highest level of perceived risk.

#### Preventive behavior

Participants reported their engagement in 10 preventive behaviors over the previous week. The ten behaviors were drawn from public health guidance at the time of the survey and included behaviors related to sanitization (e.g., wash hands), transmission control (e.g., wear mask), and social distancing (e.g., avoid visiting with others in person). The full list of behaviors can be found in Table 1.

**Table 1:** Percentage of population engaging in each preventive behavior, by political party identification, June 2020. Bolded numbers indicate a statistically significant relation between party identification and engagement in behavior

Preventive Behavior	Overall	Political Party Identification						Relation of Identification and Behavior
		Strong Republican	Weak Republican	Independent Republican	Independent Democrat	Weak Democrat	Strong Democrat	OR (95% CI)
	% population engaging in behavior	% population engaging in behavior	% population engaging in behavior	% population engaging in behavior	% population engaging in behavior	% population engaging in behavior	% population engaging in behavior	
**Avoid Shopping**	**36.3**	**29.6**	**25.4**	**30.0**	**42.9**	**38.0**	**46.2**	**1.18 (1.01, 1.39)**
**Avoid In-Person Work**	**56.6**	**36.0**	**53.4**	**60.8**	**53.9**	**60.0**	**69.0**	**1.23 (1.05, 1.44)**
Avoid Touching Face	63.0	53.9	62.5	56.1	59.2	67.9	75.4	1.14 (0.97, 1.32)
**Encourage Family to Stay Home**	**63.8**	**43.9**	**61.9**	**39.9**	**68.3**	**79.5**	**87.5**	**1.46 (1.25, 1.72)**
**Avoid Visiting with Others in Person**	**65.2**	**48.8**	**65.3**	**60.2**	**75.9**	**57.6**	**80.0**	**1.17 (1.02, 1.35)**
Disinfect Surfaces	76.1	77.8	72.0	65.1	86.3	68.0	85.8	0.98 (0.82, 1.18)
**Avoid Public Transit**	**81.3**	**68.1**	**75.5**	**73.1**	**89.0**	**84.9**	**94.2**	**1.33 (1.11, 1.60)**
**Wearing Mask**	**86.6**	**70.1**	**86.9**	**78.6**	**95.1**	**92.2**	**96.4**	**1.44 (1.14, 1.83)**
Use Hand Sanitizer	87.6	82.9	90.9	85.0	91.3	83.9	91.7	1.08 (0.87, 1.33)
**Wash Hands**	**98.0**	**100**	**100**	**99.1**	**100**	**91.8**	**97.1**	**0.53 (0.34, 0.84)**

All reported analyses control for age, education, ethnicity, gender, income, and rural/urban residence

For each behavior, participants were asked if they had done each behavior in the past 7 days (yes/no). Analyses include both dichotomous individual behavior measures for each individual behavior as well as a count of the number of the ten behaviors in which participants engaged.

### Analysis

The IPSOS KnowledgePanel survey team calculated adjusted design weights to address potential differential non-response to the survey. These adjusted design weights were calculated by assessing the distribution of US adults from the most recent fielding of the Current Population Survey. All reported analyses were conducted using STATA version 16.1 (Stata Corp., College Station, TX) and incorporated design weights to provide population representative descriptive and inferential estimates. Given that political affiliation is not independent of demographic characteristics, all reported analyses control for age, education, ethnicity, gender, income, and rural/urban residence.

To examine the relation between political affiliation and engagement in preventive behaviors, we estimated linear regression models for the overall number of preventive behaviors participants reported. The six-level party identification variable was used as a continuous predictor variable, following the ANES recommendations, and the number of behaviors reported was modelled as a continuous outcome variable. We then estimated a separate logistic regression model for each of the individual behaviors, with behavioral engagement as a dichotomous (no, yes) outcome variable and party identification as a continuous predictor variable.

To identify the relation between political affiliation and risk perception, we estimated linear regression models for each component of perceived risk and for each referent target. In each model, perceived risk (e.g., absolute risk for the average person in the us) was modelled as a continuous outcome variable and political affiliation was modelled as a continuous predictor variable.

## Results

Six hundred eighty-three adults drawn from a representative sample of the Knowledge Panel were invited to participate. The survey was completed by 410 participants (completion rate of 60% [48]). The weighted demographics of the sample, per sampling design, mirror those of the US population. Of particular importance for this paper, 17% of the population identified as strongly Republican, 10% as weakly Republican, 20% as Independent/Lean Republican, 15% as Independent/Lean Democrat, 15% as weakly Democrat, and 23% as strongly Democrat.

### Perceived risk

Individuals’ perceptions of their own personal risk, their degree of feeling fear about the possibility of infection, and their feelings of risk are reported in Table 2. As indicated by the positive slopes for the relation of political affiliation (in which higher numbers indicate more strongly Republican affiliation) and risk (where higher numbers indicate greater risk), for all three categories of risk, the sense of personal risk of infection rises as one shifts from more strongly Republican to more strongly Democratic Party identification.

**Table 2:** Perceived personal and average US risk of SARS-CoV-2 infection, by political party identification. Bolded numbers indicate a statistically significant relation between party identification and perception of risk

	Political Party Identification						Relation of Identification and Risk Perception
	Strong Republican	Weak Republican	Independent Republican	Independent Democrat	Weak Democrat	Strong Democrat	Slope (95% CI)
Self	Mean (SD)	Mean (SD)	Mean (SD)	Mean (SD)	Mean (SD)	Mean (SD)	
**Absolute Risk**	**1.76 (0.67)**	**2.05 (0.66)**	**1.82 (0.79)**	**1.97 (0.53)**	**2.16 (0.71)**	**1.94 (0.83)**	**0.084 (0.032, 0.14)**
**Fear**	**1.47 (0.63)**	**1.83 (0.76)**	**1.63 (0.62)**	**2.14 (0.91)**	**2.49 (0.95)**	**2.52 (1.04)**	**0.17 (0.12, 0.23)**
**Feelings**	**1.66 (0.81)**	**1.92 (0.72)**	**1.66 (0.67)**	**1.98 (0.64)**	**2.08 (0.84)**	**1.98 (0.76)**	**0.082 (0.031, 0.13)**
**Severity**	**2.26 (1.04)**	**2.47 (1.03)**	**2.18 (0.85)**	**2.71 (0.99)**	**2.75 (0.84)**	**2.91 (0.98)**	**0.13 (0.59, 0.20)**
Average US							
**Absolute Risk**	**2.19 (0.74)**	**2.79 (0.78)**	**2.62 (0.68)**	**3.05 (0.76)**	**3.20 (0.75)**	**3.11 (0.86)**	**0.18 (0.072, 0.18)**
**Fear**	**1.68 (0.76)**	**2.05 (0.75)**	**1.80 (0.73)**	**2.41 (0.71)**	**2.65 (0.96)**	**2.67 (0.88)**	**0.16 (0.11, 0.22)**
**Feelings**	**1.88 (0.55)**	**2.33 (0.86)**	**2.20 (0.73)**	**2.73 (0.60)**	**2.96 (0.76)**	**2.75 (0.81)**	**0.13 (0.081, 0.182)**
**Severity**	**2.34 (1.03)**	**2.65 (0.84)**	**2.49 (0.80)**	**2.99 (0.69)**	**3.04 (0.73)**	**3.24 (0.76)**	**0.13 (0.074, 0.19)**

All reported analyses control for age, education, ethnicity, gender, income, and rural/urban residence

Also reported in Table 2 are individuals’ perceptions of the average US adult’s risk, their degree of feeling fear about the possibility of infection for the average adult, and their feelings of risk for the average adult. As with personal risk, the perception of the average US adult risk of infection rises steadily as one shifts from more strongly Republican to more strongly Democratic Party identification.

Table 2 contains associations between political affiliation and perceptions of how serious/severe an infection would be for both the individual respondent and the average US adult. As with risk perception, perceptions of both own and other severity increase as party identification moves from more strongly Republican to more strongly Democratic.

### Preventive behaviors

**Fig. 1:**
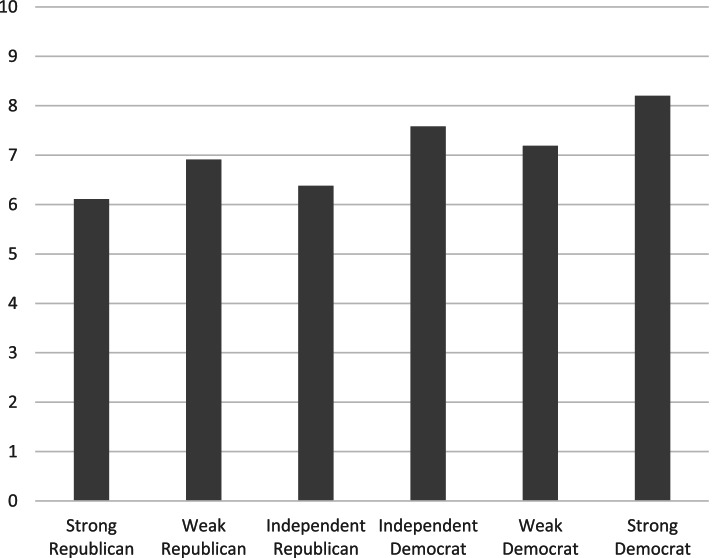
Number of Preventive Behaviors By Political Party Identification

The number of preventive behaviors participants reported engaging in as a function of party identification is presented in Fig. 1. Although respondents of all political party identifications reported some preventive behaviors, as can be seen in the figure, the number of preventive behaviors increases as party identification shifts from more strongly Republican to more strongly Democratic; linear trend b = 0.30, t(396) = 3.59, *p* < .001 (95% CI 0.14, 0.47). In follow-up analyses, both the independent-leaning Democrat and the strong Democrat categories were significantly different than the strongly Republican (both t(396) > 3.06, both *p* < 0.01).

Table 1 reports the percentage of respondents reporting engaging in each individual behavior in the past 7 days, separated by political party identification. For six of the ten preventive behaviors, engagement in the behavior became significantly more likely as one shifted from stronger identification with the Republican Party to stronger identification with the Democratic Party (e,g, 80% of strong Democrats reported avoiding in person visits, whereas only 48% of strong Republicans reported the same behavior; ORs range from 1.18 to 1.53; the measure of party affiliation ranges from strongly Republican to strongly Democratic, so ORs above 1 indicate that behavior becomes more likely as one moves toward being more strongly Democratic). The only opposite effect was for hand washing, where likelihood became significantly lower with a shift from Republican to Democratic identification (OR 0.53). There was not a significant difference for disinfecting surfaces, avoiding touching face, or use of hand sanitizers. Note that the analysis for handwashing did NOT include demographic covariates, because so few participants reported not handwashing that there were multiple missing cells for demographics/behavior combinations.

## Discussion

Political party affiliation was consistently related to the degree of risk people perceived from SARS-CoV-2 infection and COVID-19 disease. This finding was consistent across multiple components of risk perception and for both perceptions of the person’s own risk and perceptions of risk to other people. Moreover, party affiliation was associated with the number of preventive behaviors a person engaged in and, for most the individual preventive behaviors, with the probability that a given person engaged in each individual behavior.

These findings are consistent with both laypeople’s observations of differences in behavior and with press reports over the course of the pandemic. In addition, they mirror non peer-reviewed reports [49]. There has been parallel work at a community/geographical level of analysis showing that the overall political makeup of counties in the US influences the degree to which residents restricted mobility during stay at home orders [50] and engaged in social distancing [51]. Importantly, to our knowledge this paper represents the first report from a nationally-representative, probability-based sample to examine both risk perceptions and preventive behaviors, thus providing strong descriptions of the effects of partisanship on risk perceptions and preventive behaviors among the American public. In addition, the finding that partisanship affects both *beliefs* about risk and *actions* that are intended to mitigate risk highlights that complex and multifaceted ways in which public responses to the pandemic have become politicized. Also, we find that our effects extend past perceptions of one’s own risk to the little studied but, in the context of infectious disease, equally important construct of perceived risk to others. Finally, our work extends beyond previous studies focusing on single behavioral responses (e.g., social distancing [51], vaccination [52] to demonstrate a remarkably consistent partisanship effect across a range of preventive behaviors with very different levels of effort, social involvement, etc. Each of these characteristics increases confidence in the conclusion that politicization of the coronavirus pandemic and of preventive measures influences the way in which the American public’s responses to the pandemic have played out over the past several months.

Finally, although our findings are framed in the context of SARS-CoV-2/COVID-19, they are likely to extend to other scientific and public health issues that become politicized. As described in the introduction, President Trump’s anti-vaccination Twitter messages have had a demonstrable effect on childhood vaccination attitudes of his supporters [37]. Politicization of public health has occurred in the past in response to such now commonplace measures as motorcycle helmet laws, milk pasteurization, water fluoridation, and mandatory seat belt use in automobiles [53–55]. Thus, the implications of these findings extend past the current pandemic to the more general environment around science, public health, public policy, and community action to protect health and well-being.

### Implications

The current SARS-CoV-2/COVID-19 public health emergency provides a clear illustration that social factors, including political party identification and the messaging and activities of politicians and leaders from those parties, influence risk perceptions and preventive behavior.

There are, of course, multiple plausible mechanisms that might explain the effect. First, as discussed in the introduction, President Trump and other politicians directly provided messaging about the pandemic. The President’s messaging overwhelmingly focused on minimizing perceptions of risk and in some cases directly undermining public health messaging about precautionary behaviors [35]. Partisan messaging from both politicians and the media includes false risk dichotomies. For example, politicians downplayed the risk of the virus to the public amplified their negative economic impact as summarized by the President’s tweet that “WE CANNOT LET THE CURE BE WORSE THAN THE PROBLEM ITSELF”. Such sentiment was echoed in the statements of governors who resisted shutdowns even in the face of rapidly increasing virus spread. Analysis of politicians’ messaging showed that Democratic members of Congress communicated about the health-related and risk-related aspects of the pandemic both earlier and more than their Republican counterparts, who were more likely to focus on business and economy messaging [56].

Because source credibility is a key factor in interpretation of information [57, 58], the matching of more health and risk focused messaging from Democratic leaders being seen as credible by those affiliating with the Democratic party versus risk minimizing messaging from those seen as credible by those identifying as Republicans could account for the findings we report here. Consistent with this argument, recent work using statements by then candidate Trump showed that regardless of the truth or falsehood of the statement, Trump supporters more strongly believed in the statement when it was attributed to the president than when it was not [59]. When media outlets and politicians selectively communicate risk information this is likely to attenuate perceptions of risk to a greater degree among those who most trust these sources - in this case Republicans.

In addition, direct spreading of misinformation about SARS-CoV-2 infection prevention as well as COVID-19 severity and treatment has been a prominent feature of the media environment [60, 61]. Some of this has resulted in illness, injury, or death to individuals who acted based upon it [62, 63]. In the early stages of the pandemic, media outlets with a more right-wing/conservative leaning orientation discussed COVID-19 misinformation more frequently than did those outlets that were more left-wing/liberal leaning [38]. President Trump was a key spreader of this misinformation, both through his Twitter account and through amplification in traditional medial sources. The President himself was cited as an information source by 37.9% of media articles containing misinformation [64]. Such scientific misinformation is likely to undermine trust in science, and trust in science is associated with following recommendations for disease prevention [65]. Susceptibility to misinformation may be even higher when it is presented by a communicator who is aligned with the recipient’s partisan position, as is the case for Republicans for communications from President Trump, since accepting misinformation is more likely when the recipient attends to ancillary cues (like partisanship) rather than focusing on judging the accuracy of the message [66].

Finally, ideological differences or other factors may underlie both political party affiliations and risk perceptions and behavioral responses. Public health interventions are shaped and constrained by the tension between individuals’ rights and community-level goals of protecting the public health [53, 67, 68]. Ideological and political arguments between individual rights and community safety have impacted implementation of public health actions [53, 69]. In the US, more politically conservative individuals expressed lower willingness to vaccinate, a relation that is partly related to lower trust in government medical officials [70]. This tension has been seen in early responses to COVID-19, with some state and national leaders opposing public health mandates on personal liberty grounds [29]. Similar to the COVID-19 opposition, there has been a trend in anti-vaccination messaging towards more policy-focused arguments [71]. In addition, factors such as health literacy, general perceptions of health, or broader beliefs about the nature of health might differ across party lines.

Opposition to public health mandates are problematic during pandemics because policy level interventions, many of which mandate engagement in or avoidance of particular behaviors [72], have profound effects on behavior. In the context of COVID-19, key policies that may affect transmission include mandated mask wearing, closure of non-essential businesses and activities, social distancing requirements, screening for disease, and testing. The polarization of people’s beliefs about these threats resulting from politicized messaging creates barriers to coordinated public health responses to mitigate the pandemic.

## Data Availability

Data, syntax code, and survey items are available upon reasonable request from the corresponding author.
